# HCoV-229E Mpro Suppresses RLR-Mediated Innate Immune Signalling Through Cleavage of NEMO and Through Other Mechanisms

**DOI:** 10.3390/ijms26031197

**Published:** 2025-01-30

**Authors:** Xavier Martiáñez-Vendrell, Puck B. van Kasteren, Sebenzile K. Myeni, Marjolein Kikkert

**Affiliations:** Molecular Virology Laboratory, Leiden University Center of Infectious Diseases (LU-CID), Leiden University Medical Center, 2333 ZA Leiden, The Netherlands; x.vendrell@lumc.nl (X.M.-V.);

**Keywords:** coronavirus, HCoV-229E, Mpro, nsp5, 3C-like protease, interferon response, immune evasion

## Abstract

In order to detect and respond to invading pathogens, mammals have evolved a battery of pattern recognition receptors. Among these, RIG-I-like receptors (RLR) are cytosolic RNA sensors that play an essential role in the innate immune response against RNA viruses, including coronaviruses. In return, coronaviruses have acquired diverse strategies to impair RLR-mediated immune responses to enable productive infection. Viral innate immune evasion mechanisms have been well studied for highly pathogenic human coronaviruses (HCoVs), and often, these activities are thought to be linked to the severe symptoms these viruses can cause. Whether other coronaviruses, including human common cold coronaviruses, display similar activities has remained understudied. Here, we present evidence that the main protease (Mpro) of common cold HCoV-229E acts as an interferon (IFN) and NF-κB antagonist by disrupting RLR-mediated antiviral signalling. Furthermore, we show that HCoV-229E, HCoV-OC43 and MERS-CoV Mpros are able to directly cleave NEMO. We also show that HCoV-229E Mpro induces the cleavage and/or degradation of multiple other RLR pathway components, including MDA5, TBK1 and IKKε. Finally, we show that HCoV-229E infection leads to a delayed innate immune response that is accompanied by a decrease in NEMO protein levels. Our results suggest that NEMO degradation during HCoV-229E infection could be mediated, in part, by cellular degradation pathways, in addition to viral Mpro-mediated cleavage. Altogether, our research unveils innate immune evasion activities of the Mpros of low-pathogenic coronaviruses, which, despite their low pathogenicity, appear to share functionalities previously described for highly pathogenic HCoVs.

## 1. Introduction

Coronaviruses (CoVs) have developed mechanisms to modulate and adjust the cellular milieu to their advantage in order to establish a successful infection. The antiviral innate immune response, which is key to the initial control of viral infections, is one of the cellular processes coronaviruses disrupt upon cell entry [1,2]. In the context of viral infections, innate immune responses typically start upon recognition of viral nucleic acids by pattern recognition receptors (PRRs), which, in turn, activate and unfold a series of downstream signalling events that ultimately result in the activation of transcription factors that initiate the production of interferons (IFNs), IFN-stimulated genes (ISGs) and pro-inflammatory cytokines [3].

The retinoic acid-inducible gene I (RIG-I)-like receptor (RLR) pathway is one of the key innate immune signalling cascades involved in primary defence against most RNA viruses, including coronaviruses. Sensing of cytosolic viral double-stranded RNA (dsRNA), the first step in this pathway, is performed predominantly by either RIG-I or melanoma differentiation-associated gene 5 (MDA5) [3,4,5,6]. Upon the binding of dsRNA, RIG-I and MDA5 undergo conformational changes and initiate downstream signalling by means of their caspase activation and recruitment domains (CARDs), which interact with the CARD domain of the mitochondrial antiviral signalling protein (MAVS). Subsequently, MAVS oligomerizes and recruits additional innate immunity pathway components that eventually trigger the activation and nuclear translocation of two groups of transcription factors that regulate the expression of genes governing the innate immunity response: interferon regulatory factors 3 and 7 (IRF3 and IRF7) and nuclear factor kappa-light-chain-enhancer of activated B-cells (NF-κB). IRF3 and IRF7 are activated upon phosphorylation by TANK binding kinase 1 (TBK1) and I kappa B kinase epsilon (IKKε). Phosphorylated IRFs then translocate to the nucleus and activate the IFN-β promoter [7,8,9]. Alternatively, RLR activation can lead to the formation of the IKK complex, which facilitates the degradation of inhibitory molecules of the IκB family that interact with and keep NF-κB transcription factors inactive in the cytoplasm. Free NF-κB molecules translocate to the nucleus and activate the IFN-β promoter and the production of various pro-inflammatory cytokines. While these two pathways downstream of MAVS are largely independent from each other, it has been observed that the nuclear factor κB (NF-κB) essential modulator (NEMO), also known as IKKγ, is a crucial part of both routes, as it also acts as a bridging molecule upstream of TBK1/IKKε activation [10].

Because of its relevance in antiviral signalling, the RLR pathway is often the target of viral immune evasion strategies, by which viruses prevent detection of their RNA or inhibit downstream signalling by means of their viral proteins, which include viral proteases (reviewed in [11,12,13]). Coronaviruses encode two or more proteases in their genome: either one or two papain-like proteases (PLpro) and the main protease (Mpro or 3C-like protease). Their main function is that of proteolytically processing viral polyproteins at several specific sites in order to generate mature and functional viral proteins [14,15]. However, several accessory functions directed at facilitating viral replication by interfering with specific cellular processes have been attributed to coronavirus proteases. For example, the PLpros of highly pathogenic coronaviruses MERS-CoV, SARS-CoV and SARS-CoV-2 have been described as deubiquitinating (DUB) and/or deISGylating enzymes [16,17,18,19] and can modulate the antiviral innate immune response by removing either ubiquitin or ISG15 post-translational modifications from key immune proteins such as STING and IRF3 in order to disrupt downstream signalling [20,21]. Mpros of several coronaviruses have also been shown to disrupt IFN-β and/or NF-κB induction. However, most work on the interplay between viral proteases and host proteins has been done for highly pathogenic human coronaviruses, mainly SARS-CoV-2, and, to a lesser extent, for animal coronaviruses of economic relevance, such as porcine epidemic diarrhoea virus (PEDV) and porcine deltacoronavirus (PDCoV). Mpros of animal coronaviruses PEDV, PDCoV and feline infectious peritonitis virus (FIPV) have been described to act as antagonists of IFN-β response by directly cleaving relevant components in the innate immune response against viruses, including STAT2 and NEMO [22,23,24,25]. Mpros of highly pathogenic human coronaviruses SARS-CoV and SARS-CoV-2 can also cleave NEMO to inhibit IFN-β and NF-κB responses, while MERS-CoV Mpro has been shown to impair interferon signalling, although no direct substrates have been identified [26,27,28,29,30]. Generally, the evasion of host immune responses by highly pathogenic HCoVs is thought to majorly contribute to disease severity [31], yet the mechanisms by which low-pathogenic coronaviruses evade host immunity have been poorly studied. Whether the Mpros of low-pathogenic coronaviruses such as HCoV-229E are able to target and disrupt antiviral immune pathways remains unknown. Therefore, further research is needed not only to better understand the mechanisms of immune evasion of low-pathogenic coronaviruses but also to explain differences in pathogenicity between different coronaviruses.

In this work, we aimed at characterizing the effect of Mpro of the common cold, *alphacoronavirus* HCoV-229E, on the RLR signalling pathway. We provide evidence that Mpro of low-pathogenic HCoV-229E disrupts RLR-mediated antiviral innate immune signalling, an evasion mechanism previously identified for the Mpro of highly pathogenic human coronaviruses SARS-CoV, SARS-CoV-2 and MERS-CoV, as well as for Mpro of several pathogenic animal coronaviruses [23,24,25,29,32,33]. Here, we show that HCoV-229E Mpro can disrupt the RLR signalling pathway at different levels and provide data indicating that Mpro induces the cleavage of several RLR components, including NEMO. We identify specific cleavage sites in NEMO that can be cleaved by HCoV-229E Mpro, as well as by HCoV-OC43 and MERS-CoV Mpros. Finally, we show that the immune response against HCoV-229E infection is delayed and that NEMO protein levels decrease upon infection. However, the latter cannot readily be explained by Mpro-directed cleavage of NEMO. Altogether, these results uncover new insights on the manipulation of host antiviral innate immune responses by HCoV-229E, HCoV-OC43 and MERS-CoV.

## 2. Results

### 2.1. The Mpro of HCoV-229E Dampens the IFN-β and NF-κB Responses

Several viral proteases can disrupt cellular antiviral innate immune responses by targeting different elements involved in either viral sensing or downstream signalling. However, it is not known whether the Mpro of low-pathogenic human coronaviruses, including HCoV-229E, can also disrupt IFN-β induction. To study this, we used a mammalian expression vector encoding the Mpro of HCoV-229E to assess the effect of its ectopic expression in HEK293T, in which IFN-β induction was primed by transient transfection of a vector coding for a constitutively active form of RIG-I, i.e., RIG-I(2CARD). Activation of the RLR pathway results in IRF3 phosphorylation, consequent oligomerization and nuclear translocation, and interaction with CREB binding protein (CBP) and/or p300 to activate IFN-β expression [7,34,35].

**Figure 1:**
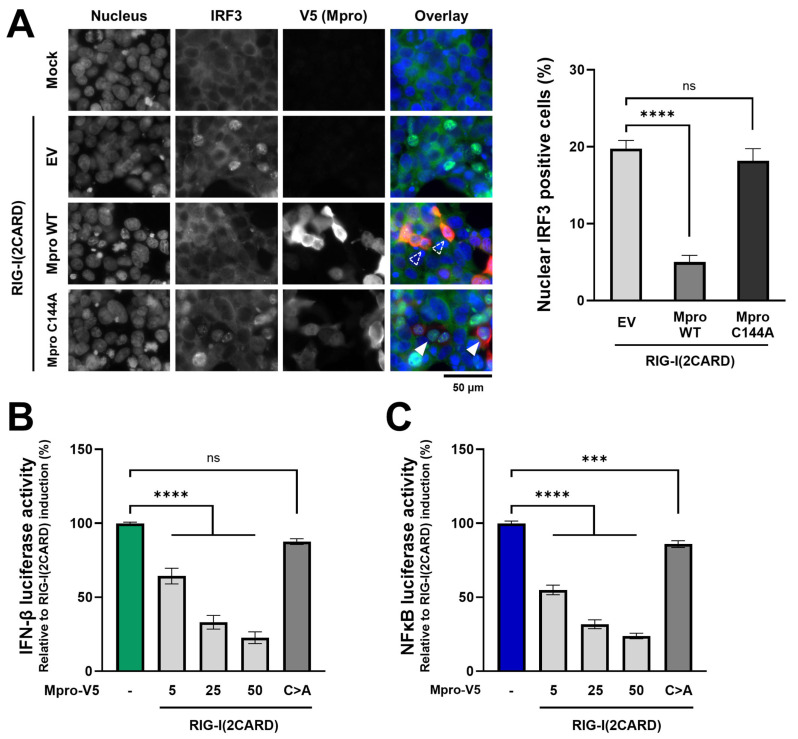
**HCoV-229E Mpro expression blocks IRF3 nuclear translocation and IFN-β and NF-κB induction.** (**A**) HEK293T cells seeded in 24-well clusters containing coverslips were mock-transfected or transfected with a plasmid coding for RIG-I(2CARD) (100 ng/well) in combination with an empty vector (EV), a plasmid encoding V5-tagged HCoV-229E Mpro WT or Mpro C144A. At 16 hpt, cells were fixed and stained with antibodies against IRF3 (green) and V5 (red), and nuclei were counterstained with Hoechst dye (blue). Dashed empty arrows indicate cells expressing Mpro and cytosolic IRF3, and solid white arrows indicate cells expressing Mpro and nuclear IRF3. (**B**) HEK293T cells in 24-well plates were co-transfected with a combination of plasmids encoding the IFN-β-luciferase reporter (50 ng/well), renilla luciferase as control for transfection efficiency (5 ng/well), RIG-I(2CARD) (25 ng/well), and HCoV-229E Mpro WT (5, 25 or 50 ng/well) or Mpro C144A (50 ng/well). At 16 hpt, luciferase activity was measured. (**C**) HEK293T cells were co-transfected with a combination of plasmids encoding the NF-κB-luciferase reporter (50 ng/well), renilla luciferase as control for transfection efficiency (5 ng/well), RIG-I(2CARD) (25 ng/well), and HCoV-229E Mpro WT (5, 25 or 50 ng/well) or Mpro C144A (50 ng/well). At 16 hpt, luciferase activity was measured. Data are shown as triplicates obtained in one of three independent experiments that yielded similar results. Differences between groups were assessed by one-way ANOVA. Mean ± SEM is shown. ns, not significant; ***, *p* < 0.0001; ****, *p* < 0.00001.

First, we evaluated the translocation of IRF3 upon expression of RIG-I(2CARD) in HEK293T cells in the absence or presence of HCoV-229E Mpro. As shown in Figure 1A, transfection of a plasmid encoding RIG-I(2CARD) led to IRF3 nuclear translocation in about 20% of cells. The percentage of nuclear IRF3-positive cells was not altered upon expression of HCoV-229E catalytic mutant Mpro, in which the active site cysteine (C) residue was replaced by an alanine (A) (Mpro C144A), but it was significantly reduced in cells co-expressing Mpro WT. Next, we assessed the effect of Mpro on IFN-β induction using a dual luciferase IFN-β reporter assay. Results showed that the Mpro of HCoV-299E inhibited RIG-I(2CARD)-induced IFN-β production in a dose-dependent manner (Figure 1B). Expression of catalytically inactivated mutant HCoV-229E Mpro (C144A) also led to a small reduction in IFN-β luciferase activity, but this was not statistically significant. Finally, we also addressed whether Mpro can disrupt RLR-mediated NF-κB induction. For this, NF-κB reporter-mediated luciferase activity was assessed in HEK293T cells in the absence or presence of HCoV-229E Mpro. Similarly to the results observed in the dual luciferase IFN-β reporter assay, expression of Mpro WT led to a reduction in luciferase activity in a dose-dependent manner, while the expression of Mpro C144A only had a limited effect on NF-κB induction (Figure 1C). These data suggest that HCoV-229E Mpro can block IFN-β induction by preventing nuclear translocation of active IRF3 and disrupt the induction of NF-κB and that these inhibitory activities are strongly dependent on the catalytic activity of Mpro.

### 2.2. Ectopic Expression of HCoV-229E Mpro Induces NEMO Cleavage by Means of Its Catalytic Activity

**Figure 2:**
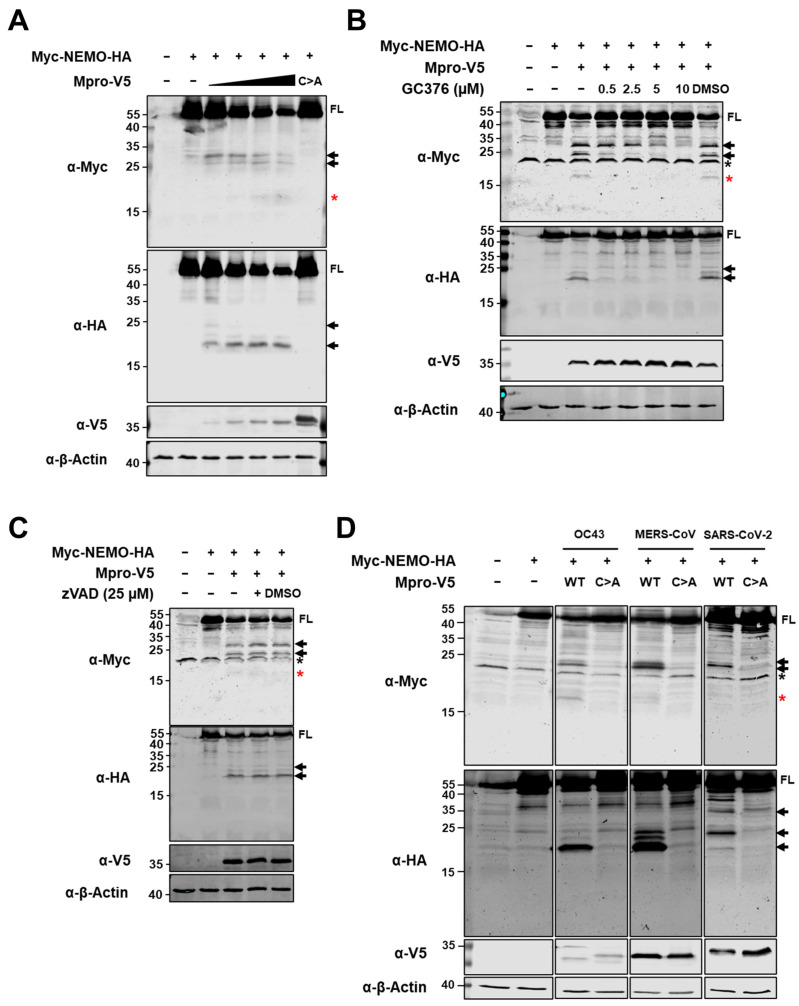
**The Mpros of HCoV-229E, HCoV-OC43 and MERS-CoV cleave NEMO by means of their catalytic activity.** (**A**) HEK293T cells were co-transfected with a plasmid coding for human Myc-NEMO-HA (2 µg/well) and increasing amounts of a V5-tagged HCoV-229E WT Mpro mammalian expression plasmid (0.25, 0.5, 1 and 2 µg/well) or catalytic mutant Mpro (2 µg/well). (**B**) Myc-NEMO-HA (2 µg/well) and V5-tagged HCoV-229E WT Mpro (2 µg/well) mammalian expression plasmids were co-transfected in HEK293T cells, and cells were treated with increasing concentrations of Mpro inhibitor GC376. (**C**) HEK293T cells were left untreated or were treated with 25 µM zVAD for 2 h prior to transfection and were then co-transfected with the Myc-NEMO-HA plasmid (2 µg/well) and the V5-HCoV-229E Mpro plasmid. (**D**) HEK293T cells were transfected with a combination of plasmids coding for double-tagged NEMO and WT Mpro of either HCoV-OC43, MERS-CoV or SARS-CoV-2 (the latter as a positive control). (**A**–**D**) At 24 h post-transfection (hpt), cells were lysed, and protein lysates were immunoblotted for Myc, HA, V5 and β-actin. Immunoblots are representative of at least three independent experiments. FL, full-length; WT, wild type; C>A, catalytic mutant Mpro; solid black arrows indicate cleaved fragments identified by immunoblotting; black asterisks indicate unspecific bands; red asterisks indicate less abundant cleaved fragments.

For several RNA viruses encoding 3C or 3C-like proteases, it has been reported that protease-associated disruption of antiviral innate immune responses occurs by protease-mediated cleavage of the NEMO adaptor protein [23,24,25,26,27,36,37,38,39]. To study whether NEMO can also be targeted for proteolytic processing by Mpro of HCoV-229E, we first co-expressed WT or C144A Mpro of HCoV-229E with N-terminally, Myc-tagged and C-terminally, HA-tagged human NEMO in HEK293T cells. Immunoblotting analysis revealed a decrease in full-length (FL) NEMO, as well as the appearance of both N-terminal and C-terminal NEMO fragments when NEMO was co-expressed with WT Mpro but not with the co-expression of Mpro C144A (Figure 2A). Further, we co-expressed Myc-NEMO-HA with HCoV-229E Mpro in the absence or presence of increasing concentrations of coronavirus Mpro inhibitor GC376 (0.5–10 µM). Increasing concentrations of GC376 rescued the presence of FL Myc-NEMO-HA and blocked the appearance of NEMO fragments in the presence of HCoV-229E Mpro (Figure 2B). Next, we considered whether HCoV-229E Mpro-induced cleavage of NEMO could be caspase-dependent, as NEMO has also been reported as a substrate of caspases [40]. However, treatment with pan-caspase inhibitor z-VAD-fmk did not inhibit the cleavage of NEMO in the presence of HCoV-229E Mpro (Figure 2C). Finally, we also investigated whether NEMO is cleaved by the Mpro of low-pathogenic HCoV-OC43, as well as the Mpro of highly pathogenic human coronavirus MERS-CoV. Since cleavage of NEMO by the Mpro of SARS-CoV-2 has recently been shown [26,27], we co-expressed NEMO and SARS-CoV-2 Mpro as a positive control. Figure 2D shows that NEMO is cleaved in the presence of HCoV-OC43 and MERS-CoV WT Mpros and confirms previous findings suggesting that the Mpro of SARS-CoV-2 cleaves NEMO [26,27,28]. Altogether, these data indicate that the Mpro of HCoV-229E, as well as the Mpros of HCoV-OC43, MERS-CoV and SARS-CoV-2, cleaves NEMO by means of its catalytic activity.

### 2.3. HCoV-229E Mpro Cleaves NEMO at Glutamine Residues 83, 205 and 231

**Figure 3:**
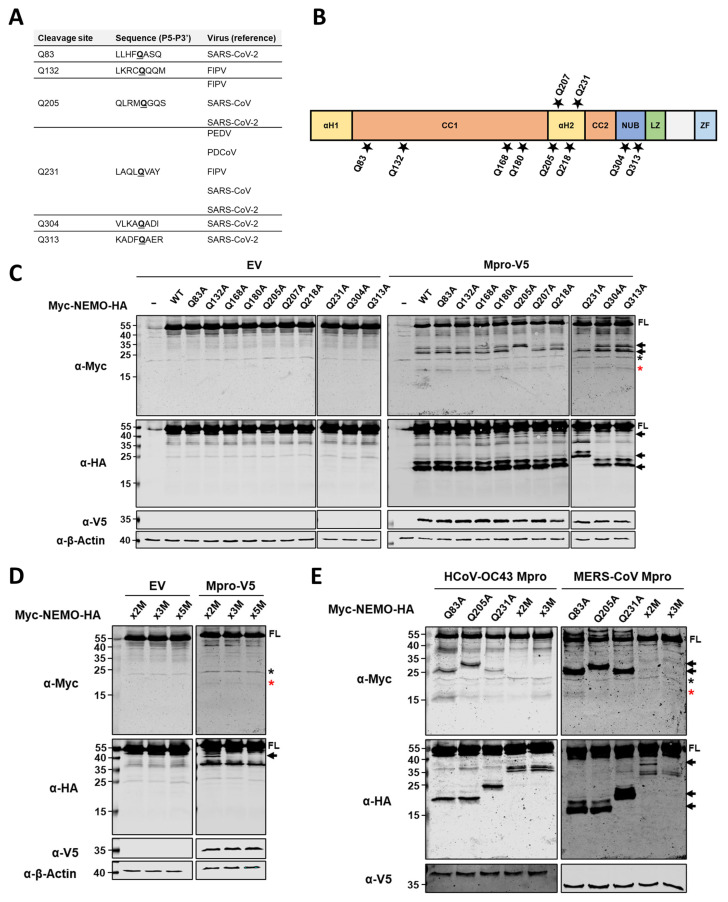
**Identification of HCoV-229E Mpro cleavage sites within NEMO.** (**A**) Previously identified cleavage sites within NEMO for PEDV [23], PDCoV [24], FIPV [25], SARS-CoV and SARS-CoV-2 [26,27]. (**B**) Schematic representation of NEMO protein in which the glutamine residues mutated into an alanine are indicated by black stars. From N-terminal to C-terminal: αH1, Helical domain 1; CC1, Coiled coil 1; αH2, Helical domain 2; CC2, Coiled coil 2; NUB, NEMO ubiquitin binding; LZ, Leucin zipper; ZF, Zinc finger. (**C**) HEK293T cells were transfected with plasmids coding for double-tagged NEMO single mutants or with a combination of a plasmids for the expression of the different NEMO mutants and V5-HCoV-229E Mpro. (**D**) Plasmids coding for NEMO double mutant (x2A, Q205A + Q231A), triple mutant (x3A, Q83A + Q205A + Q231A) or quintuple mutant (x5A, Q83A + Q205A + Q231A + Q304A + Q313A) were transfected alone or in combination with V5-HCoV-229E Mpro. (**E**) The indicated NEMO mutants were co-expressed in HEK293T cells either alone or together with the V5-tagged Mpros of HCoV-OC43 or MERS-CoV. (**C**–**E**) At 24 hpt, cells were lysed, and protein lysates were immunoblotted for Myc, HA, V5 and β-actin. Immunoblots are representative of at least three independent experiments. FL, full-length; WT, wild type; C>A, catalytic mutant Mpro; solid black arrows indicate cleaved fragments identified by immunoblotting; black asterisks indicate unspecific bands; red asterisks indicate less abundant cleaved fragments.

We next examined the location of Mpro cleavage sites within human NEMO. Previous studies on Mpro substrate specificity reported a preference for a small hydrophobic residue at P4, a glutamine (Q) at the P1 position and a small amino acid residue at P1′ [41,42,43]. Additionally, several cleavage sites within NEMO have already been described for the Mpros of other animal and human coronaviruses, including Q83, Q132, Q205, Q231, Q304 and Q313 (Figure 3A). Based on this information, we constructed a series of NEMO mutants in which individual glutamine residues were replaced by alanine residues (Figure 3B). Plasmids coding for the NEMO mutants listed in Figure 3B were transfected alone (with an empty vector, EV) or co-transfected with a plasmid coding for HCoV-229E Mpro WT in HEK293T cells. At 24 hpt, cells were harvested, and protein lysates were analysed by immunoblotting. As shown in Figure 3C, we observed that upon co-expression of HCoV-229E Mpro with NEMO mutants Q205A and Q231A, at least one cleaved fragment was no longer present, while for all other NEMO mutants, no differences in the pattern of cleavage products were clearly discernible as compared to the WT NEMO and Mpro condition.

Next, we constructed a NEMO double mutant (x2A) containing Q-to-A substitutions in residues 205 and 231. Co-expression of this NEMO double mutant with HCoV-229E Mpro revealed an additional C-terminal (HA-tagged) product of about 40 kDa (Figure 3D). This fragment was no longer observed when an additional mutation (Q83A) was introduced to the NEMO x2A to generate NEMO x3A. These results suggest that Mpro preferentially cleaves at residues Q205 and Q231 within NEMO, but it can also cleave at additional sites when these glutamine residues are mutated and no longer available for cleavage. To address whether previously identified cleavage sites at positions Q304 and Q313 for SARS-CoV-2 Mpro are also potential cleavage sites for HCoV-229E Mpro that are difficult to detect when other cleavage sites are available, we introduced Q304A and Q313A into NEMO x3AM to generate NEMO x5A [26]. Co-expression of NEMO x5A with the Mpro of HCoV-229E did not show differences compared to NEMO x3A + Mpro, suggesting either that HCoV-229E Mpro does not cleave at Q304 and/or Q313 or that cleavage at these sites could occur at a very low frequency, not allowing for the detection of the resulting cleavage products by immunoblotting.

Finally, we investigated whether cleavages at the identified residues (Q83, Q205 and Q231) are also found for the Mpros of HCoV-OC43 and MERS-CoV by co-expressing NEMO single mutants for these residues, NEMO x2A or NEMO x3A with either HCoV-OC43 Mpro or MERS-CoV Mpro. As shown in Figure 3E, Q205A and Q231A mutations abrogated Mpro-mediated cleavage at these positions for both HCoV-OC43 and MERS-CoV Mpros. Similarly to our previous observations for Mpro of HCoV-229E, co-expression of NEMO x2A with Mpros of HCoV-OC43 and MERS-CoV yielded another C-terminal (HA-tagged) fragment that was absent for NEMO x3A, suggesting that the Mpros of both HCoV-OC43 and MERS-CoV also cleave NEMO at Q83.

### 2.4. Besides by Cleavage of NEMO, HCoV-229E Mpro Likely Abrogates NF-κB Induction Through Other Mechanisms

**Figure 4:**
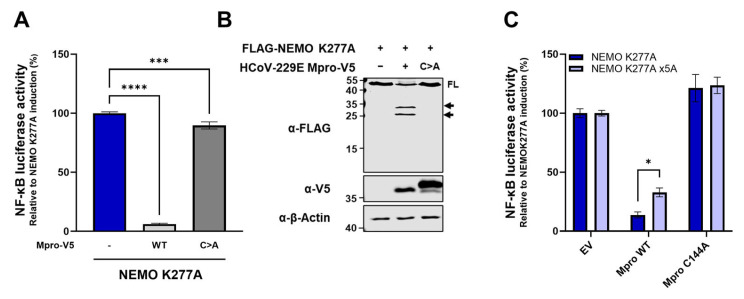
**HCoV-229E Mpro-uncleavable NEMO barely recovers NF-κB induction in the presence of Mpro.** (**A**) Plasmids coding for NF-κB-luciferase reporter (50 ng/well), renilla luciferase as a control for transfection efficiency (5 ng/well), FLAG-NEMO K277A (400 ng/well), and HCoV-229E Mpro WT (50 ng/well) or Mpro C144A (50 ng/well) were co-transfected in HEK293T cells in 24-well plates. At 16 hpt, cells were lysed, and luciferase activity was measured. (**B**) HEK293T cells were co-transfected with mammalian expression vectors for FLAG-NEMO K277A and HCoV-229E Mpro WT or C144A. At 24 hpt, cells were lysed, and protein lysates were immunoblotted for FLAG, V5 and β-actin. (**C**) HEK293T cells were co-transfected with plasmids coding for NF-κB-luciferase reporter (50 ng/well), renilla luciferase as a control for transfection efficiency (5 ng/well), FLAG-NEMO K277A or FLAG-K277A x5A (400 ng/well), and HCoV-229E Mpro WT (50 ng/well) or Mpro C144A (50 ng/well). At 16 hpt, cells were lysed, and luciferase activity was measured. Dual-luciferase reporter assays were performed as triplicates and repeated at least three times. Differences between groups were assessed by one-way ANOVA (panel (**A**)) or two-way ANOVA (panel (**C**)). Immunoblots are representative of two independent experiments. Mean ± SEM is shown. FL, full-length; WT, wild type; C>A, catalytic mutant Mpro; solid black arrows indicate cleaved fragments identified by immunoblotting; EV, empty vector; *, *p* < 0.01; ***, *p* < 0.0001; ****, *p* < 0.00001.

To investigate whether HCoV-229E Mpro cleavage of NEMO can disrupt RLR-mediated induction of IFN-β and NF-κB, we used a constitutive active NEMO mutant (NEMO K277A) that has previously been used to induce the activation of NF-κB, as well as transcription of IFN-β [24,25,36,44,45]. NEMO K277A was expressed in HEK293T in both dual luciferase NF-κB and IFN-β reporter assays, either alone or in the presence of HCoV-229E WT or C144A. Expression of NEMO K277A did not induce IFN-β reporter-mediated luciferase activity, but it did induce NF-κB reporter-mediated luciferase activity. Expression of HCoV-229E Mpro WT significantly reduced luciferase activity, while Mpro C144A only had a minor effect on NF-κB reporter-mediated luciferase activity (Figure 4A). Co-expression of NEMO K277A with HCoV-229E was accompanied by a reduction in full-length NEMO and cleavage of NEMO in two major cleavage products (Figure 4B). To assess whether an Mpro-resistant NEMO mutant could recover NF-κB reporter-mediated luciferase activity, we next introduced Q-to-A substitutions at positions 83, 205, 231, 304 and 313 in NEMO K277A (NEMO K277A x5A). Luciferase activity induced by NEMO K277A x5A significantly decreased in the presence of HCoV-229E Mpro but showed a slight but statistically significant recovery compared to that in the NEMO K277A and Mpro condition (Figure 4C). These results suggest that NF-κB activity is disrupted by the catalytic activity of HCoV-229E Mpro and that such disruption is only partially mediated by cleavage of NEMO.

### 2.5. HCoV-229E Mpro Disrupts the RLR Signalling Pathway Downstream of NEMO

**Figure 5:**
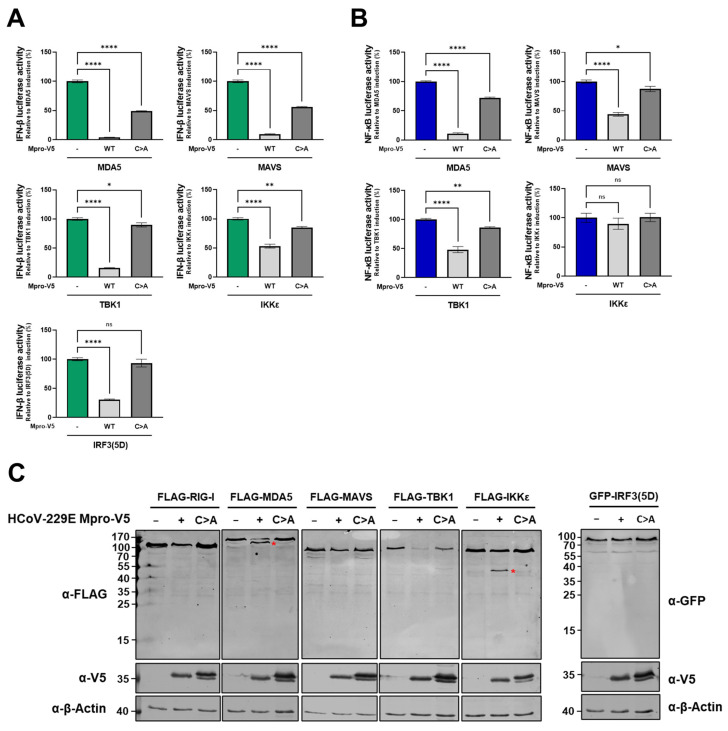
**The Mpro of HCoV-229E disrupts the RLR signalling pathway at multiple levels.** (**A**) HEK293T cells were co-transfected with plasmids encoding the IFN-β-luciferase reporter (50 ng/well), renilla luciferase as a control for transfection efficiency (5 ng/well), and HCoV-229E Mpro WT (50 ng/well) or Mpro C144A (50 ng/well), together with a plasmid expressing MDA5 (25 ng/well), MAVS (25 ng/well), TBK1 (50 ng/well), IKKε (50 ng/well) or IRF3(5D) (50 ng/well). At 16 hpt, cells were lysed, and luciferase activity was measured. (**B**) HEK293T cells were co-transfected with a combination of plasmids encoding the NF-κB-luciferase reporter (50 ng/well); renilla luciferase as a control for transfection efficiency (25 ng/well); HCoV-229E Mpro WT (50 ng/well) or Mpro C144A (50 ng/well); and MDA5 (25 ng/well), MAVS (25 ng/well), TBK1 (50 ng/well) or IKKε (50 ng/well). At 16 hpt, cells were lysed, and luciferase activity was measured. (**C**) Plasmids coding for FLAG-tagged RLR ligands RIG-I (2 μg/well), MDA5 (2 μg/well), MAVS (2 μg/well), TBK1 (2 μg/well), IKKε (2 μg/well) or GFP-IRF3 (2 μg/well) were co-transfected with plasmids coding for HCoV-229E Mpro WT or C144A. At 24 hpt, cells were lysed, and protein lysates were immunoblotted for FLAG or GFP, V5 and β-actin. Data are shown as triplicates obtained in one of three independent experiments that yielded similar results. Differences between groups were assessed by one-way ANOVA. Mean ± SEM is shown. Immunoblots are representative of two independent experiments. FL, full-length; EV, empty vector; WT, wild type; C>A, catalytic mutant Mpro; red asterisks indicate cleavage products; ns, not significant; *, *p* < 0.01; **, *p* < 0.001; ****, *p* < 0.00001.

Since we observed that Mpro can disrupt NF-kB signalling independently of NEMO cleavage, we aimed at determining whether there are other proteins in the RLR signalling pathway that can be targeted by Mpro to hamper either IFN-β induction or NF-κB activity. First, we investigated the effect of HCoV-229E Mpro expression on the IFN-β promoter activation mediated by pathway components acting at different levels within the RLR pathway, including MDA5, MAVS, TBK1, IKKε and IRF3(5D) (a constitutively active form of IRF3) [7]. Induction of IFN-β reporter-mediated luciferase activity was significantly inhibited in the presence of HCoV-229E Mpro for all tested RLR pathway components and partially recovered in co-expression with Mpro C144A (Figure 5A). Similarly, HCoV-229E Mpro overexpression also inhibited RLR-mediated activation of NF-κB upon induction with MDA5, MAVS and TBK1 but not upon induction with IKKε (Figure 5B). These results suggest that overexpression of Mpro potentially inhibits RLR signalling at any level within the pathway, both upstream and downstream of NEMO.

To further explore whether additional RLR pathway components other than NEMO can be targeted by HCoV-229E Mpro, HEK293T cells were co-transfected with plasmids encoding FLAG-tagged RIG-I, MDA5, MAVS, TBK1 or IKKε or GFP-tagged IRF3 in combination with an empty vector or a plasmid encoding HCoV-229E Mpro. Interestingly, Mpro overexpression led to the cleavage of MDA5 and IKKε, as well as to decreased TBK1 protein levels (Figure 5C). Altogether, these data depict a complex scenario in which HCoV-229E Mpro could be acting redundantly and simultaneously on different RLR components to inhibit the innate antiviral immune response.

### 2.6. The Effect of Mpro on IFN-β and NF-κB Induction Is Independent of Mpro-Mediated Toxicity

**Figure 6:**
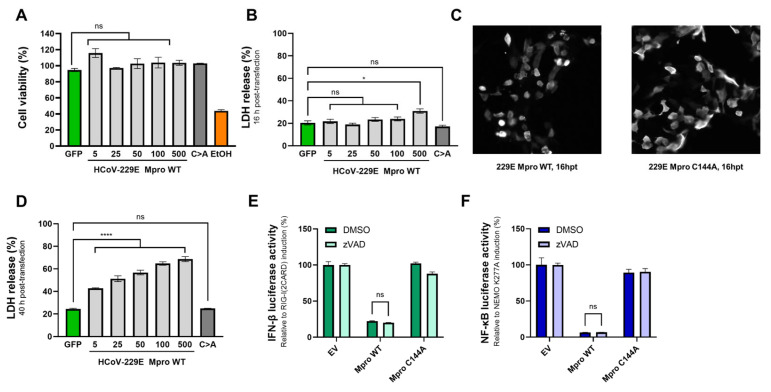
**The impairment of IFN-β and NF-κB induction by HCoV-229E Mpro is not attributed to Mpro-mediated cytotoxicity.** (**A**,**B**) HEK293T cells in 24-well plates were transfected with a plasmid encoding eGFP (negative control, 500 ng/well), increasing amounts (5, 25, 50, 100 and 500 ng/well) of a plasmid coding for HCoV-229E Mpro WT or a plasmid encoding HCoV-229E Mpro C144A (500 ng/well) or treated with ethanol (EtOH) as a positive control for cell death. At 16 hpt, cell viability (**A**) and/or lytic cell death (**B**) were assessed by MTS assay and LDH release assay, respectively. (**C**) HEK293T cells were transfected with a plasmid coding for V5-tagged HCoV-229E Mpro WT (50 ng/well) or a plasmid coding for Mpro C144A (50 ng/well). At 16 hpt, cells were fixed and stained with an antibody against V5. (**D**) HEK293T cells were transfected with a plasmid encoding eGFP (negative control, 500 ng/well), increasing amounts (5, 25, 50, 100 and 500 ng/well) of a plasmid coding for HCoV-229E Mpro WT or a plasmid encoding HCoV-229E Mpro C144A (500 ng/well). At 40 hpt, lytic cell death was assessed. (**E**) HEK293T cells treated with DMSO or fmk-zVAD (25 µM) were co-transfected with a combination of plasmids encoding the IFN-β-luciferase reporter (50 ng/well), renilla luciferase as control for transfection efficiency (5 ng/well), RIG-I(2CARD) (25 ng/well), and HCoV-229E Mpro WT (50 ng/well) or Mpro C144A (50 ng/well). At 16 hpt, luciferase activity was measured. (**F**) HEK293T cells treated with DMSO or fmk-zVAD (25 µM) were co-transfected with a combination of plasmids encoding the NF-κB-luciferase reporter (50 ng/well), renilla luciferase (5 ng/well), NEMO K277A (400 ng/well), and HCoV-229E Mpro WT (50 ng/well) or Mpro C144A (50 ng/well). At 16 hpt, luciferase activity was measured. Experiments were performed once (**A**,**E**,**F**) or twice (**B**,**C**) as triplicates. Differences between groups were assessed by one-way ANOVA (panels (**A**,**B**,**D**)) or two-way ANOVA (panels (**E**,**F**)). Mean ± SEM is shown. LDH, lactate dehydrogenase; EV, empty vector; WT, wild type; C>A, catalytic mutant Mpro; ns, not significant; *, *p* < 0.01; ****, *p* < 0.00001.

So far, we have observed Mpro-mediated disruption of IFN-β and NF-κB induction upon priming the RLR signalling pathway with different components within this pathway, both upstream and downstream of NEMO. To exclude the possibility that the overall inhibitory effect caused by Mpro is an indirect effect of Mpro-mediated cell toxicity, as it has been previously reported that the overexpression of various coronaviruses Mpros can cause cell toxicity and cell death [46], we performed an overexpression assay in HEK293T cells. Equal amounts of the plasmid coding for HCoV-229E Mpro used in previous dual-luciferase reporter assays (5, 25 and 50 ng/well), as well as two greater amounts (100 and 500 ng/well), were transfected, and at 16h post transfection, we used MTS and LDH release assays to assess cell viability and lytic cell death, respectively. Transfection of 5 to 100 ng/well of the HCoV-229E Mpro-encoding expression vector did not lead to a decrease in cell viability (Figure 6A) or an increase in lytic cell death (Figure 6B) as compared to cells transfected with a GFP expression vector, and only a slight but significant increase in lytic cell death was observed in cells transfected with 500 ng/well of plasmid coding for Mpro WT. The expression of the catalytic mutant Mpro (500 ng/well) did not cause a decrease in cell viability or an increase in lytic cell death. Although no cell death was measured at 16 hpt with the amounts of Mpro expression vector used in dual-luciferase reporter assays (5–50 ng/well), we observed differences in cell morphology between cells expressing Mpro WT and cells expressing Mpro C144A (Figure 6C). At 16 hpt, cells expressing Mpro WT (50 ng/well) presented with cell shrinkage, a typical characteristic of apoptotic cell death. Additionally, Mpro expression did cause cell death at 40 hpt in a dose-dependent manner as measured by LDH release assay (Figure 6D), probably indicating that HCoV-229E Mpro triggers cell death-inducing events that can eventually lead to cell death. Considering these observations, we next performed both IFN-β and NF-κB dual-luciferase reporter assays in the presence of pan-caspase inhibitor zVAD, which has been described as preventing apoptotic cell death in a variety of cell lines. Treatment of Mpro WT-expressing cells with zVAD did not prevent the inhibitory effect of Mpro on the induction of IFN-β and NF-κB (Figure 6E,F). These data indicate that, although the overexpression of HCoV-229E Mpro can lead to cell death, the previously observed Mpro-mediated inhibition of IFN-β and NF-κB induction is not attributable to major cell death events such as activation of proapoptotic caspases.

### 2.7. HCoV-229E Infection Leads to a Delayed Antiviral Innate Immune Response and Reduced NEMO Protein Expression Levels

**Figure 7:**
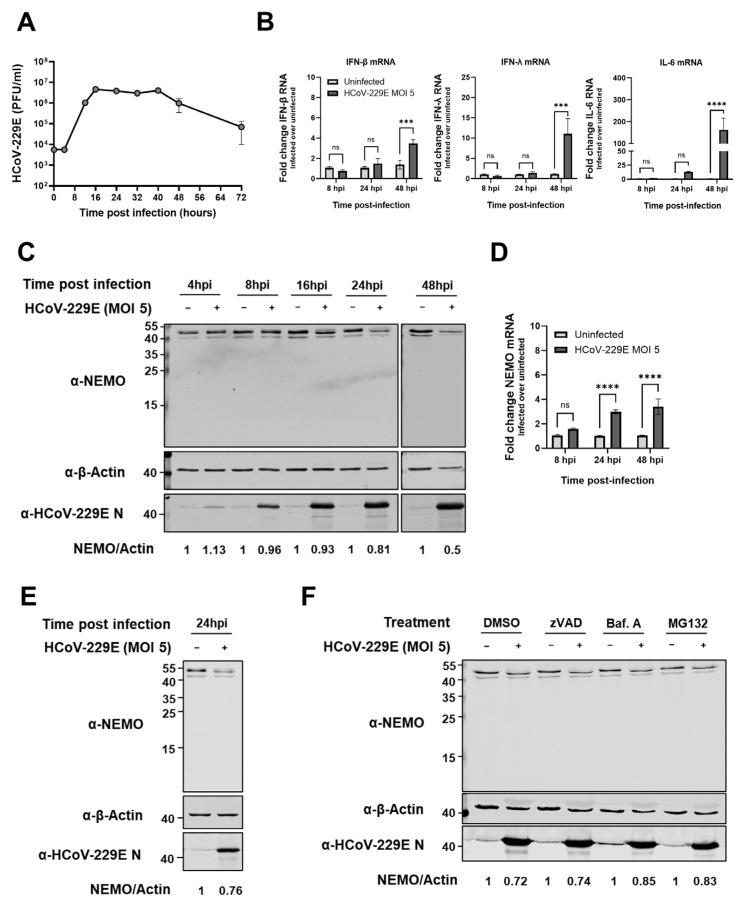
**HCoV-229E infection leads to delayed innate immune responses and to decreased NEMO protein levels.** (**A**) Huh7 cells were infected with HCoV-229E at an MOI of 5, supernatants were harvested at the indicated time points and HCoV-229E infectious virus particles were quantified by plaque assay. (**B**) Huh7 cells were infected with HCoV-229E at an MOI of 5; then, 8, 24 and 48 hpi, cell lysates were harvested, and IFN-β, IFN-λ and IL-6 mRNA levels were determined by RT-qPCR. (**C**) Huh7 cells were infected with HCoV-229E at an MOI of 5, and protein lysates were collected at the indicated time points and analysed by immunoblotting for NEMO, HCoV-229E nucleocapsid (N) protein and β-actin. (**D**) Huh7 cells were infected with HCoV-229E at an MOI of 5; then, 8, 24 and 48 hpi, cell lysates were harvested, and NEMO mRNA levels were determined by RT-qPCR. (**E**) BEAS-2B cells were infected with HCoV-229E at an MOI of 5, and at 24 hpi, protein lysates were collected and analysed by immunoblotting for NEMO, HCoV-229E nucleocapsid protein and β-actin. (**F**) Huh7 cells were mock-infected or infected with HCoV-229E at an MOI of 5 and treated with vehicle control (DMSO), zVAD (25 µM) or bafilomycin A1 (100 nM) for 24 h or with MG132 (20 µM) for 12 h (from t = 12–24 hpi). At 24 hpi, protein lysates were collected and analysed by immunoblotting for NEMO, HCoV-229E nucleocapsid protein and β-actin. Experiments were performed as triplicates and repeated at least twice. Differences between groups were assessed by one-way ANOVA. Immunoblots are representative of two (**F**) or more (**C**,**E**) independent experiments. ns, not significant; ***, *p* < 0.0001; ****, *p* < 0.00001. Mean ± SEM is shown. Amounts of NEMO are normalized by β-actin and expressed relative to the corresponding controls and are indicated below Western blot panels.

Next, we sought to investigate whether the results observed upon Mpro overexpression would, to some extent, recapitulate in the context of HCoV-229E infection. To determine the infection dynamics, we first assessed the growth overtime of HCoV-229E in Huh7 cells infected at a multiplicity of infection (MOI) of 5 (Figure 7A). Next, we measured the expression of immune genes at early (8 h) and late (24 and 48 h) time points post infection in Huh7 cells infected with HCoV-229E. HCoV-229E induced both IFN-β and IFN-λ transcription in infected Huh7 cells, but only at a late stage during infection, and a similar pattern was observed for the NF-κB-regulated IL-6 gene (Figure 7B). We then hypothesized that the delayed immune response signature could be partially mediated by Mpro cleavage of endogenous NEMO during infection. Immunoblotting analysis showed that full-length NEMO protein levels decreased in infected Huh7 cells as compared to uninfected cells in a time-dependent fashion, but no cleaved fragments were identified (Figure 7C). Analysis of NEMO mRNA expression levels ruled out the possibility that protein levels were decreased due to lower transcription levels of NEMO in infected cells (Figure 7D). Disappearance of NEMO during HCoV-229E infection was also observed in bronchial epithelial cell line BEAS-2B, but again, NEMO fragments were not observed (Figure 7E). In light of these data, we next asked whether NEMO could be degraded during infection by means of other degradation mechanisms. It has been reported that NEMO can be cleaved by apoptotic caspases [40]. Additionally, NEMO can undergo both lysosomal and proteasomal degradation in different scenarios, including virus infection [47,48,49,50,51]. Therefore, we treated HCoV-229E-infected cells with pan-caspase inhibitor zVAD, autophagosome-lysosome fusion inhibitor bafilomycin A1 or proteasome inhibitor MG132 and analysed NEMO protein levels at 24 hpi. Immunoblotting analysis showed that zVAD treatment did not influence NEMO protein levels as compared to DMSO-treated (mock) cells, suggesting that caspases do not play a role in the degradation of NEMO. Upon inhibition of the lysosomal and proteasomal degradation pathways, NEMO protein levels also decreased as compared to those in uninfected cells, but to a slightly lower extent (Figure 7F). These data suggest that cellular degradation pathways may play a role in the reduction of NEMO protein levels, while not ruling out the possibility that NEMO could still be partially cleaved by HCoV-229E Mpro in the context of infection.

## 3. Discussion

In this study, we present evidence that the Mpro of common cold HCoV-229E acts as an IFN agonist by disrupting RLR-mediated antiviral signalling, which aligns with previous results for highly pathogenic human coronaviruses and animal coronaviruses [22,23,24,25,29,30,32]. We report that Mpros of HCoV-229E, HCoV-OC43 and MERS-CoV are able to induce cleavage of NEMO and that HCoV-229E Mpro is also able to induce the cleavage and/or degradation of multiple other RLR pathway components, including MDA5, TBK1 and IKKε. During HCoV-229E infection, NEMO protein levels decrease, and the innate immune response is delayed. Altogether, our work unveils possible innate immune evasion activities of the Mpros of HCoV-229E and HCoV-OC43, which, despite their low pathogenicity, appear to share functionalities previously described for human coronaviruses of high pathogenicity, such as MERS-CoV and SARS-CoV-2 [26,27,29,30].

Antagonism of interferon-mediated innate immune response by viral proteases appears to be a common immune evasion strategy among a wide range of RNA viruses [11]. Here, we explored the effect of HCoV-229E Mpro on the induction of IFN-β and NF-κB activation. We first observed that in cells primed with RIG-I(2CARD), the expression of HCoV-229E Mpro WT blocked nuclear translocation of IRF3 (Figure 1A). Similar findings have been recently reported for the Mpros of highly pathogenic SARS-CoV-2 and MERS-CoV [30,32,52]. For SARS-CoV-2, it has been suggested that the expression of Mpro promotes the degradation of IRF3, but it remains unknown whether such degradation happens as a result of the direct action of Mpro on IRF3 or through an indirect mechanism [52]. Alternatively, the Mpro of MERS-CoV has been shown to prevent IRF3 nuclear translocation without affecting either protein expression or phosphorylation of IRF3, but an interaction was identified between MERS-CoV Mpro and IRF3, which could explain the impairment of IRF3 nuclear translocation [30]. Considering that IRF3 nuclear translocation is inhibited in the presence of Mpro from distinct coronaviruses, in future studies, it would be interesting to explore the exact mechanism by which Mpro does so in the context of infections. Upon phosphorylation, IRF3 binds CREB binding protein (CREBBP), and the complex translocates into the nucleus, where it promotes the transcription of interferons [7]. The disruption of IRF3 nuclear translocation by Mpro could explain the decrease in IFN-β expression we observed using an IFN-β dual-luciferase reporter assay (Figure 1B). This finding aligns with previous research in which the expression of Mpro of other coronaviruses has been reported to decrease IFN-β induction using similar assays [23,24,25,27,29,30,32,33,52,53]. However, recent studies that screened most SARS-CoV-2 proteins for their effect on IFN-β induction did not find Mpro to inhibit it [54,55,56,57]. Further, our results indicate that the expression of HCoV-229E Mpro also disrupted the activation of the NF-κB-dependent promoter in our assay (Figure 1C), similarly to what we recently reported for the Mpro of MERS-CoV [29]. Another group has shown that the Mpros of animal-infecting coronaviruses PEDV and PDCoV also inhibit NF-κB induction [23,24]. Additionally, Mpro of SARS-CoV-2 blocks nuclear translocation of p65, a component of the NF-κB complex, upon IL-1β treatment or during infection with Sendai virus (SeV) [26,52]. On the other hand, reports suggest that the expression of SARS-CoV and SARS-CoV-2 Mpros activates the NF-κB pathway [58,59]. Such discrepancies in the current literature could be explained by methodological variations but raise questions regarding Mpro’s involvement in the disruption of the interferon and NF-κB responses. Future studies in the context of viral infections are needed to more accurately assess the impact Mpros of coronaviruses have on the host’s innate immune response.

The innate immune evasion mediated by coronavirus Mpros is mostly attributed to the direct cleavage of specific innate immune proteins. In particular, the Mpro of PDCoV cleaves signal transducer and activator of transcription 2 (STAT2) to impair the JAK-STAT pathway and reduce the production of interferon-stimulated genes (ISGs) [22]. Furthermore, the Mpro of SARS-CoV-2 has been described as cleaving histone deacetylase 2 (HDAC2), the function of which is required to enhance the transcriptional elongation of ISGs [60]. Recently, the Mpro of MERS-CoV has been shown to induce the cleavage and degradation of MAVS, an important adaptor protein in the RLR signalling pathway [29]. Finally, the Mpros of several highly pathogenic human and animal coronaviruses can cleave NEMO, a key molecule for the activation of both the NF-κB and IRF3 pathways [23,24,25,26,27,28]. NEMO has also been described as a substrate of other viral chymotrypsin-like proteases, including the 3C protease of picornaviruses foot and mouth disease virus (FMDV) and hepatitis A virus (HAV) [38,45], nsp4 of arteriviruses such as equine arterivirus (EAV) and porcine reproductive and respiratory disease virus (PRRSV) [37,44] and the 3C-like protease of norovirus [39]. Since cleavage of NEMO by 3C and 3C-like proteases, including the Mpros of several coronaviruses, is well documented in the literature, we first asked whether the Mpro of HCoV-229E could also cleave NEMO. We found that NEMO cleavage was induced not only by the Mpro of HCoV-229E but also by the Mpros of HCoV-OC43 and MERS-CoV (Figure 2), expanding the known range of coronaviruses that can target NEMO by means of Mpro activity. Our results reveal that the Mpros of these three viruses cleave NEMO at glutamine residues 83, 205 and 231 (Figure 3). Cleavage at Q231 has been observed for all coronaviruses whose Mpros are known to cleave NEMO; therefore, this site seems to be a major target for these viral proteases [23,24,25,26,27,28]. The Q83 residue has also been identified as a cleavage site for SARS-CoV-2 Mpro [26], and both SARS-CoV-2 and FIPV Mpros can cleave NEMO at Q205 [25,26,27]. Interestingly, Q205 is also a cleavage site within NEMO for other viral chymotrypsin-like proteases, such as nsp4 of equine arterivirus (EAV) and the 3C-like protease of norovirus [37,39]. We did not find Q304 and Q313 to be cleavage sites for the Mpros of HCoV-229E, HCoV-OC43 or MERS-CoV. These sites were identified for the Mpro of SARS-CoV-2 by mass spectrometry upon incubation of recombinant NEMO and Mpro proteins [26]. We possibly missed these cleavage sites due to a lack of sensitivity of our method. Alternatively, it is possible that such cleavages do not occur in an overexpression system in cells such as the one used in this work, as these sites could be masked by interactions of NEMO with other proteins. Finally, a recent study shows that the Mpros of SARS-CoV and SARS-CoV-2 can cleave NEMO at glutamic acid 152 (E152) [27]. In some experiments in which we looked at cleavage of NEMO by HCoV-229E Mpro, we could detect a faint N-terminal NEMO product of approximately 15–20 kDa that could potentially result from cleavage at E152, although we did not confirm it (Figure 3C, indicated with a red asterisk). For years, it has been considered that a Q residue at the P1 position of the cleavage site is a requirement for the efficient hydrolysis of a substrate by Mpro, but recent research suggests that coronavirus Mpros can also cleave after a histidine (H), even if at lower frequencies [61]. In the future, it would be interesting to further explore whether coronavirus Mpros can accommodate amino acid residues other than Q and H at the P1 position, including E.

In light of the relevant role NEMO plays in the RLR signalling pathway as an adaptor protein, we hypothesized that the antagonistic effect of HCoV-229E Mpro on IFN-β and NF-κB induction we initially observed could be explained by the Mpro-mediated cleavage of NEMO. As previously mentioned, we could not induce IFN-β reporter activity after transfection of a plasmid coding for NEMO K277A. The substitution of lysine 270 for an alanine in murine NEMO was reported to confer NEMO the capacity to strongly activate NF-κB [62]. The corresponding substitution in human NEMO (K277A) was shown to also induce NF-κB, although to a minor extent [36,62]. In our hands, NEMO K277A activated the NF-κB-dependent promoter, but the co-expression of HCoV-229E abrogated activation and resulted in the cleavage of NEMO (Figure 4A,B). Surprisingly, NF-κB activation was only minorly restored in the presence of Mpro when we used a NEMO K277A mutant harbouring Q-to-A substitutions at all Q sites known to be hydrolysed by coronavirus Mpros (Q83, Q205, Q231, Q304 and Q313) (Figure 4C). These results indicate that, although cleavage of NEMO by HCoV-229E Mpro contributes to the impairment of NF-κB activation, Mpro acts at other levels to disrupt the NF-κB signalling pathway, at least in the context of this assay. This was confirmed by the observation that Mpro could disrupt RLR-mediated activation of NF-κB upon induction with ligands not only upstream of NEMO but also downstream, such as TBK1. Similarly, Mpro expression impaired IFN-β reporter activation when IFN-β activation was induced with the expression of TBK1, IKKε and constitutively active IRF3, all of which are positioned downstream of NEMO in the signalling pathway. These data are in contrast with previous research showing that the Mpros of PEDV, PDCoV and FIPV do not disrupt IFN-β activation downstream of NEMO [23,24,25]. Although these are animal coronaviruses, this observation was made in HEK293T in which human RLR ligands (TBK1 and/or IRF3(5D)) were expressed to induce IFN-β reporter activity. Because of that, a possible explanation would be that the Mpros of these viruses, which have evolved in different hosts, do not target certain components in the human RLR pathway or other human proteins that could play a role in IFN-β induction, although these Mpros can still hydrolyse human NEMO. Nonetheless, for PEDV it was observed that cells expressing PEDV Mpro-uncleavable NEMO (NEMO Q231A) only partially restored IFN-β reporter activity upon infection with SeV when Mpro was present [23], which would still point to additional Mpro-mediated effects in the disruption of innate immune responses other than the cleavage of NEMO, warranting further investigation.

Interestingly, several RNA viruses act redundantly on innate immune signalling pathways, either by means of the same viral protease targeting different factors in the cascade or by the action of two or more proteases encoded in the viral genome acting on the same pathway. We observed that HCoV-229E Mpro expression also led to the cleavage of dsRNA sensor MDA5 and serine/threonine kinase IKKε (Figure 5C). To the best of our knowledge, cleavage of any of these two proteins has never been identified for coronaviruses before. However, MDA5 is cleaved in poliovirus-infected cells, presumably upon activation of caspases [63]. Additionally, several picornavirus proteases have been described to cleave MDA5 during infection, including the leader protease of FMDV and the 2A protease of coxsackievirus B3 (CVB3) [64,65]. The 3C protease of CVB3, as well as that of the murine picornavirus Theilovirus, can also cleave MDA5 upon overexpression of both the protease and MDA5 in cells [64,66]. In future studies, we aim to further characterize HCoV-229E Mpro-mediated cleavage of MDA5, as well as that of IKKε, which, so far, has not been described for any viral protease. HCoV-229E Mpro not only cleaved different RLR ligands, but its expression also drastically reduced TBK1 protein levels without generating detectable TBK1 fragments, suggesting that TBK1 undergoes degradation in the presence of Mpro. TBK1 has been identified as a substrate of several E3 ligases that promote its proteasomal degradation, subsequently downregulating interferon responses [67,68,69,70,71]. Interestingly, expression of porcine Sapovirus (PSaV) NS6 protease, a 3C-like protease, leads to proteasomal degradation of TBK1 [72]. Whether Mpros of HCoV-229E and other coronaviruses can induce the degradation of TBK1 by means of their Mpros is therefore of interest for further research.

So far, we have observed that the ectopic expression of HCoV-229E Mpro clearly disrupts IFN-β and NF-κB induction and induces the cleavage of NEMO, as well as the cleavage or degradation of other RLR pathway components. Next, we asked whether, in the context of HCoV-229E infection, we could observe the suppression and/or delay of innate immune responses, matching the effects we investigated. HCoV-229E induced both IFN-β and IFN-λ transcription in infected Huh7 cells but only at a late stage during infection, and a similar observation was made for the NF-κB-regulated IL-6 gene (Figure 7B). Interestingly, the delayed induction of innate immune genes was accompanied by a decrease in NEMO protein levels that could not be explained by a reduction in NEMO gene transcription (Figure 7C,D). However, we were not able to detect NEMO fragments in the context of viral infection. Similar to our observation, previous reports could not identify NEMO fragments upon PEDV or PDCoV infection, despite the clear reduction in full-length NEMO in infected cells [23,24]. The absence of NEMO fragments in infected cells could be explained by the low stability of these fragments and their rapid degradation. Alternatively, full-length NEMO could be (at least partially) degraded by cellular degradation machineries such as the autophagy lysosomal or the proteasomal degradation pathways. NEMO has been shown to undergo lysosomal degradation after associating with p47, a key component for Golgi membrane fusion, during TNF-α or IL-1 stimulation [47]. Murine cytomegalovirus can also promote lysosomal degradation of NEMO by means of a virus-encoded protein and subsequently inhibit NF-κB activation [48]. On the other hand, both human and murine NEMO can be targeted to the proteasome in the context of bacterial and viral infections by E3 ligases MARCH2 and TRIM29, respectively, while E3 ligase TRIM13 has been shown to modulate NEMO ubiquitination and turnover to control the activity of the IKK complex upon TNF stimulation [49,50,51]. In our hands, treatment of HCoV-229E-infected Huh7 cells with autophagosome inhibitor bafilomycin A and proteasome inhibitor MG132 partially rescued NEMO during HCoV-229E infection but not completely (Figure 7F). Another possible explanation for the lower levels of NEMO in infected cells is the translational shutoff mediated by coronavirus non-structural protein 1 (nsp1). It has been shown that HCoV-229E and other *alphacoronaviruses* can inhibit mRNA translation by means of nsp1, even if in a mechanistically different manner than that of most *betacoronaviruses*, including SARS-CoV-2 [73,74,75,76]. Altogether, our results suggest that the decrease in full-length NEMO during coronavirus infections is at least not fully dependent on Mpro-mediated cleavage of NEMO. A more thorough investigation on the loss of NEMO in coronavirus-infected cells is therefore needed to better understand the role of NEMO, as well as to characterize Mpro-associated immune evasion activities in more detail in the context of viral infection.

In summary, our research identifies HCoV-229E Mpro as an antagonist of the RLR-mediated innate immune response. This disruptive effect relies on Mpro’s catalytic activity, by which it induces cleavage of NEMO, and cleavage or degradation of additional RLR pathway components both up- and downstream of NEMO. Additionally, our results suggest that immune evasion activities previously attributed to Mpros of highly pathogenic coronaviruses are also present in Mpros of human common cold coronaviruses. These insights highlight the need for future comparative studies between coronaviruses of different pathogenicity that aim at elucidating whether Mpro plays a role in disease severity. Finally, our data suggest that other forces may also target NEMO and cause the protein to disappear over the course of viral infection.

## 4. Materials and Methods

### 4.1. Cell Culture and Virus Infections

HEK293T cells (ATCC, cat.no. CRL-3216) were cultured in Dulbecco’s modified Eagle medium (DMEM, Gibco, Thermo Fisher Scientific, Waltham, MA, USA) supplemented with 10% foetal calf serum (FCS, Bodinco, Alkmaar, The Netherlands), 100 units/mL penicillin and 100 units/mL streptomycin (Cat.no. P4458-100ML, Sigma-Aldrich, St. Louis, MO, USA). Huh7 (a kind gift from Dr. Ralf Bartenschlager, Heidelberg University, Germany) and BEAS-2B (a kind gift from Prof. Andrew G. Bowie, Trinity Biomedical Sciences Institute, Trinity College, Dublin, Ireland) cells were maintained in DMEM with 8% FCS, 100 units/mL penicillin, 100 units/mL streptomycin, 2mM L-glutamine (PAA Laboratories, Pasching, Austria) and non-essential amino acids (NEAA, PAA Laboratories). All cells were cultured at 37 °C in 5% CO_2_ incubators.

### 4.2. Virus Infections

Infection with HCoV-229E (GenBank accession number NC_002645.1, [77]) was performed in DMEM containing 2% FCS, 2 mM L-glutamine, 100 units/mL penicillin, 100 units/mL streptomycin and NEAA at 33 °C in 5% CO_2_ (infection medium). Cells were inoculated for 1 h, washed two times with phosphate-buffered saline (PBS), refreshed with infection medium and incubated at 33 °C. All experiments with HCoV-229E were performed in a biosafety level 2 (BSL-2) laboratory.

### 4.3. Plaque Assays

Huh7 cells (4 × 10^5^ cells/well) were seeded in 2 mL cell culture medium in 6-well plates and incubated overnight at 37 °C in 5% CO_2_. The day after, 10-fold serial dilutions of supernatant samples were prepared in infection medium. Cell culture medium was removed, and 500 μL of serial dilutions was added to the wells. Cells were incubated at 37 °C with gentle rocking for 1 h, after which the virus inoculum was aspirated and 2 mL/well overlay medium (1.2% Avicel, 1% antibiotics, 2% FCS, and 50 mM HEPES in DMEM) was added. Cells were incubated at 33 °C for 4 days, when overlay medium was removed and cells were fixed with 1 mL/well 3.7% formaldehyde. After fixation, cells were stained using 1.25% *w/v* crystal violet, and plaques were manually counted to determine the infectious virus titre in the supernatant samples.

### 4.4. Plasmids Used for Transfections

The following plasmids have been previously described: pLuc-IFN-β [78], pcDNA3.1-Mpro (HCoV-229E)-V5 wild-type and C144A [79], pcDNA3.1-Mpro (MERS-CoV)-V5 wild-type and C148A [29], pcDNA3.1-Mpro (HCoV-OC43)-V5 wild-type and C145A [79], pcDNA3.1-V5-Mpro (SARS-CoV-2) wild-type and C145A [79], pEF-RIG-I(2CARD) [80], pcDNA3.1-FLAG-MAVS [64], pEGFP-C1-IRF3(5D) [7], and pcDNA-eGFP [81]. pLuc-NF-κB was a kind gift from Dr. Adolfo Garcia-Sastre (Icahn School of Medicine at Mount Sinai, New York, NY, USA). pEF-MDA5 was a kind gift from Dr. F. van Kuppeveld (Faculty of Veterinary Medicine, Utrecht, The Netherlands). pcDNA3.1-TBK1 was a kind gift from Dr. J. Hiscott (Istituto Pasteur Italia—Fondazione Cenci Bolognetti, Rome, Italy). IKKε-FLAG was a kind gift from Dr. M. Ressing (LUMC, Leiden, The Netherlands). pRL-TK-expressing renilla luciferase was purchased from Promega (Leiden, The Netherlands). A pcDNA3.1 vector containing the sequence for His-Xpress-NEMO was used as a basis for cloning the NEMO sequence to a pcDNA3.1 vector in frame with an N-terminal Myc tag and a C-terminal HA tag (pcDNA3.1-Myc-NEMO-HA). The NEMO sequence was also cloned into a pcDNA3.1 expression vector (V79020, Thermo Fisher Scientific) in frame with an N-terminal FLAG tag. pcDNA3.1-Myc-NEMO-HA and pcDNA3.1-FLAG-NEMO were used as templates for site-directed mutagenesis using the QuickChange^TM^ strategy to change glutamine (Q) at positions 83, 132, 168, 180, 205, 207, 218, 231, 304 and 313 into alanine (A), as well as lysine (K) at position 277 into A. The sequences of the primers used for site-directed mutagenesis is available upon request. Primers used for plasmid construction and site-directed mutagenesis were designed in Geneious version 10.2.6 (Biomatters, Auckland, New Zealand). All constructs were sequence-verified by Sanger sequence analysis performed at the Leiden Genome Technology Center (Leiden, The Netherlands).

### 4.5. Immunoblotting Analysis

Protein lysates were obtained upon cell lysis using a mild lysis buffer (20 mM Tris-HCl pH 7.4, 135 mM NaCl, 1% Triton X-100, 10% glycerol). Cell lysates were centrifuged for 30 min at 15,000× *g* and at 4 °C. Supernatants were collected, mixed with 2× Laemmli sample buffer (LSB, 250mM tris-base pH 6.8, 4% SDS, 20% glycerol, 10mM DTT, and 0.01% bromophenol blue) at 1:1 ratio and boiled at 96 °C for 5 min. Protein lysates were subjected to SDS-PAGE using either 15% or 12.5% SDS-PAGE gels, then blotted onto either nitrocellulose 0.2 µm (GE10600004, Amersham, Zwijndrecht, The Netherlands) or polyvinylidene difluoride 0.2 µm (GE10600022, Amersham, UK) membranes by semi-dry blotting using a Trans-blot Turbo system (Biorad, Hercules, CA, USA). Membranes were blocked in 5% dried milk powder in PBS with 0.05% Tween-20 (PBST) for 1 h, followed by overnight incubation with the corresponding primary antibody in 5% dried milk powder in PBST at 4 °C. The following primary antibodies were used for the detection of tagged proteins in co-transfection experiments: mouse anti-V5 at 1:2500 (clone 2F11F7, Thermo Fisher Scientific), rabbit anti-HA at 1:3000 (ab137838, Abcam, Cambridge, UK), rabbit anti-Myc at 1:2500 (ab9106, Abcam) and mouse anti-FLAG (F3165, Sigma-Aldrich). A rabbit anti-NEMO antibody at 1:5000 (ab137363, Abcam) was used to detect endogenous NEMO, and a rabbit polyclonal serum raised in-house against HCoV-229E nucleocapsid protein at 1:5000 was used to detect HCoV-229E N. β-actin was used as a loading control and detected using mouse anti-β-actin at 1:10000 (A5316, Sigma-Aldrich). Secondary IRDye^®^ 680RD goat α-mouse and IRDye^®^ 800CW goat α-rabbit antibodies were used for fluorescence detection with an Odyssey scanner (Licor, Lincoln, NE, USA). The band intensity of immunoblotting images was quantified using ImageJ software (National Institutes of Health, Bethesda, MD, USA).

### 4.6. Dual-Luciferase Reporter Assay

HEK293T cells in 24-well plates (1.5 × 10^5^ cells/well) were co-transfected using polyethylenimine (PEI 25 K, Polysciences, Warrington, PA, USA) with reporter plasmid IFN-β-Luc (50 ng/well), an internal control plasmid expressing renilla luciferase (5 ng/well), plasmids coding for innate immune response-inducing proteins (RIG-I(2CARD), 25 ng/well; MDA5, 25 ng/well; MAVS, 25 ng/well; TBK1, 50 ng/well; IKKε, 50 ng/well; IRF3(5D), 50 ng/well; TRAF6, 50 ng/well; or NEMO K277A, 400 ng/well), and plasmids expressing the Mpro of HCoV-229E or MERS-CoV (5 to 50 ng/well). A pcDNA3.1-Empty construct (empty vector) was added to transfection mixes to equalize the total amount of DNA transfected for each combination of plasmids. At 16 h post transfection (hpt), cells were lysed, and cell lysates were used to determine luciferase activity following the manufacturer’s instructions (Dual-Luciferase Reporter Assay System, Promega, Leiden, The Netherlands) using an EnVision multiplate reader (PerkinElmer, Waltham, MA, USA). Each assay was repeated independently at least three times, and each separate experiment was performed in triplicate. Luciferase activity was calculated by normalizing firefly luciferase activity to renilla luciferase activity, and the results were expressed relative to the corresponding control sample.

### 4.7. Immunofluorescence Microscopy

Transfected HEK293T cells grown on glass coverslips in 24-well plates (1.5 × 10^5^ cells/well) were fixed with 3% paraformaldehyde (PFA) in PBS. Coverslips were then washed with PBS and permeabilized with 0.2% Triton X-100 in PBS for 10 min at room temperature (RT). After permeabilization, cells were washed with PBS and incubated with a rabbit anti-IRF3 antibody (D6I4C, Cell Signaling Technologies, Danvers, MA, USA) (1:300) and a mouse anti-V5 antibody (clone 2F11F7, Thermo Fisher Scientific, Waltham, MA, USA) (1:500) in 5% FCS in PBS for 1 h at RT. Next, coverslips were washed with PBS and subsequently incubated with secondary antibodies goat anti-rabbit alexa-488 antibody (Thermo Fisher/Invitrogen, Waltham, MA, USA) (1:300) and donkey anti-mouse-Cy3 antibody (Jackson ImmunoResearch, West Baltimore Pike West Grove, PA, USA) (1:300) in 5% FCS in PBS in the dark for 1 h at room temperature. Hoechst 33,258 (Thermo Fisher) (1:100) was used for nucleus staining. Coverslips were embedded on glass slides with ProLong glass antifade mountant (P36984, Thermo Fisher). Images were acquired using a Leica DM6B fluorescence microscope and analysed with Leica Application Suite X software (version 3.8.1.26810, Leica Microsystems, Wetzlar, Germany).

### 4.8. RNA Isolation and RT-qPCR

To determine the expression of NEMO and that of genes related to the immune response (IFN-β, IFN-λ and IL-6) in Huh7 and BEAS-2B cells during HCoV-229E infection, cells were collected in Tripure Isolation Reagent (Sigma-Aldrich), intracellular RNA was isolated following the manufacturer’s instructions and RT-qPCR was performed. Briefly, 1 μg of RNA was reverse-transcribed into complementary DNA (cDNA) using RevertAid H minus reverse transcriptase (Thermo Fisher Scientific) and random hexamers (Promega). Gene expression was determined by RT-qPCR using iQ SYBR Green supermix (BioRad, Veenendaal, The Netherlands) on a CFX384 Touch Real-Time PCR Detection System (BioRad) with the following program: 3 min at 95 °C, 30 s at 60 °C, 40 cycles of 10 s at 95 °C, 10 s at 60 °C and 30 s at 72 °C, followed by 10 s at 95 °C and melt curve analysis using a temperature gradient from 60 °C to 95 °C with 0.5 °C increments. Primers targeting mRNAs encoding human β-actin (Forward (Fwd): 5′-AGGCACCAGGGCGTGAT-3′; Reverse (Rev): 5′-GCCCACATAGGAATCCTTCTGAC-3′), human IFN-β (Fwd: 5′-GTCACTGTGCCTGGACCATA-3′; Rev: 5′-GCTTGAAGCAATTGTCCCGT-3′), human interleukin 6 (IL-6) (5′-AGAGGCACTGGCAGAAAACAAC-3′; Rev: 5′-AGGCAAGTCTCCTCATTGAATCC-3′), human NEMO (5′-AAGAGCCAACTGTGTGAGATG-3′; Rev: 5′-TTCGCCCAGTACGTCCTGA-3′) and human IFN-λ-I (Fwd: 5′-GTGACTTTGGTGCTAGGCTTG-3′; Rev: 5′-GCCTCAGGTCCCAATTCCC-3′) were used. Relative gene expression was calculated using the comparative Ct (ΔΔCt) method, with β-actin as housekeeping gene, and expressed as fold change over the corresponding mock or uninfected sample.

### 4.9. Cell Death Assays

The viability of HEK293T cells expressing HCoV-229E Mpro was assessed at 16 hpt by MTS assay using a CellTiter 96 aqueous nonradioactive cell proliferation kit (Promega). Lytic cell death was indirectly studied at 16 and 40 hpt by measuring lactate dehydrogenase (LDH) release in cell culture medium using a CytoTox 96 nonradioactive cytotoxicity assay kit (G1780, Promega, Leiden, The Netherlands) according to the manufacturer’s instructions. For both assays, absorbance was measured at 490nm with an EnVision multiplate reader (PerkinElmer, Waltham, MA, USA).

### 4.10. Statistical Analysis

Data obtained by dual-luciferase reporter assay and RT-qPCR were analysed with GraphPad Prism 9.3.1. Data are presented as mean ± standard error of the mean (SEM). One-way or two-way ANOVA was used to assess statistical significance, and *p*-values of <0.05 were considered statistically significant.

## Data Availability

Data are contained within the article. Negative data regarding the induction of IFN-β luciferase with NEMO mutant K277A are available upon request.
